# Increased Prescribing of Attention-Deficit/Hyperactivity Disorder Medication and Real-World Outcomes Over Time

**DOI:** 10.1001/jamapsychiatry.2025.1281

**Published:** 2025-06-25

**Authors:** Lin Li, David Coghill, Arvid Sjölander, Honghui Yao, Le Zhang, Ralf Kuja-Halkola, Isabell Brikell, Paul Lichtenstein, Brian M. D’Onofrio, Henrik Larsson, Zheng Chang

**Affiliations:** 1 Department of Medical Epidemiology and Biostatistics, Karolinska Institutet, Stockholm, Sweden; 2 Departments of Pediatrics and Psychiatry, University of Melbourne, Melbourne, Victoria, Australia; 3 Murdoch Children’s Research Institute, Melbourne, Victoria, Australia; 4 Department of Global Public Health and Primary Care, University of Bergen, Bergen, Norway; 5 Department of Biomedicine, Aarhus University, Aarhus C, Denmark; 6 Department of Psychological and Brain Sciences, Indiana University, Bloomington, Indiana; 7 School of Medical Sciences, Örebro University, Örebro, Sweden

## Abstract

**Importance:**

The prescription of attention-deficit/hyperactivity disorder (ADHD) medications has risen substantially in many countries over the last 20 years. However, whether the real-world benefits of ADHD medications change with increased prescription rates within a society remains unknown.

**Objective:**

To examine whether the associations between ADHD medications and real-world outcomes (self-harm, unintentional injury, traffic crashes, and crime) change as prescription rates rise.

**Design, Setting, and Participants:**

This study used a self-controlled case series design. It was a population-based study using Swedish National Registers that included individuals who used ADHD medications in Sweden between 2006 and 2020. Data were analyzed from October 2023 to November 2024.

**Exposure:**

ADHD medication use.

**Main Outcomes and Measures:**

Rates of self-harm, unintentional injury, traffic crashes, and crime during medicated vs nonmedicated periods. The associations between ADHD medication and these real-world outcomes were examined across 3 time periods, 2006 to 2010, 2011 to 2015, and 2016 to 2020, during which ADHD medication prevalence increased from 0.6% to 2.8%.

**Results:**

There were 247 420 individuals identified (99 361 females [40.2%] and 148 059 males [59.8%]) aged 4 to 64 years in Sweden who used ADHD medications between 2006 and 2020. ADHD medication was consistently associated with lower risks for self-harm (incidence rate ratio [IRR] ranged from 0.77; 95% CI, 0.73-0.81 to 0.85; 95% CI, 0.82-0.88), unintentional injury (IRR ranged from 0.87; 95% CI, 0.84-0.89 to 0.93, 95% CI, 0.91-0.95), traffic crashes (IRR ranged from 0.71; 95% CI, 0.67-0.77 to 0.87; 95% CI, 0.83-0.91), and crime (IRR ranged from 0.73; 95% CI, 0.71-0.75 to 0.84; 95% CI, 0.82-0.85) across different age groups, sexes, and over time. However, the associations between ADHD medication use and lower risks of unintentional injury (*P* value for trend < .01), traffic crashes (*P* value for trend < .01), and crime (*P* value for trend < .01) appear to weaken over time as prescription rates increased. Changes in age and sex distribution of individuals receiving ADHD medication did not fully explain the weakening trend for unintentional injury and traffic crashes.

**Conclusions and Relevance:**

In this study, ADHD medication remained associated with reduced risks of several serious real-world outcomes. However, the magnitude of these associations appears to have decreased alongside rising prescription rates over time. Thus, it is important to regularly evaluate medication use in different patient populations.

## Key Points

**Question:**

Do the real-world benefits of attention-deficit/hyperactivity disorder (ADHD) medications in reducing adverse outcomes change as prescription rates increase in the population?

**Findings:**

In this nationwide study of 247 420 ADHD medication users in Sweden from 2006 to 2020, ADHD medication use was consistently associated with lower risks of self-harm, unintentional injury, traffic crashes, and crime, while some of these associations weakened as prescriptions rates increased.

**Meaning:**

These results demonstrated that ADHD medications were consistently associated with reduced risks of several serious real-world outcomes, but these associations appear to weaken alongside shifts in the patient population, highlighting the need for ongoing review of treatment guidelines.

## Teaser

This study examines whether the associations between attention-deficit/hyperactivity disorder medications and real-world outcomes (self-harm, unintentional injury, traffic crashes, and crime) change as prescription rates rise.

## Introduction

Attention-deficit/hyperactivity disorder (ADHD) is the most prevalent neurodevelopmental disorder, affecting approximately 5.3% of youths and 2.5% of adults worldwide.^1^ Individuals with ADHD present increased risks for a wide array of psychiatric and somatic conditions, serious functional outcomes, and premature death.^1^ This makes the delivery of safe and effective treatments, that improve both the core symptoms and related impairments, a key public health issue. Randomized clinical trials have demonstrated that ADHD medications are effective in reducing core ADHD symptoms and large observational studies further indicate they are associated with lower risks of real-world outcomes, including injuries, crime, transport crashes, suicide attempts, and unnatural-cause mortality.^2,3,4^

Until recent years, in many countries, the administrative prevalence of diagnosed ADHD was much lower than the estimated epidemiological prevalence.^5^ However, the diagnosis of ADHD and prescription of ADHD medication has increased substantially worldwide over the last decade.^6,7^ A recent study reported an annual rise of 9.7% in ADHD medication use across 64 countries between 2015 and 2019.^7^ Sweden has seen a particularly sharp increase in ADHD medication use over the years. From 2006 to 2020, ADHD medication use in children increased nearly 5-fold, from 0.6% to 2.8%, and more than 10-fold in adults, from 0.1% to 1.3% during this period, positioning Sweden among countries with the highest rates of ADHD medication prescription globally. Similar trends are seen in other European countries.^6,7^ Research suggests that these increases in prescription are not a response to actual increases in ADHD prevalence, but rather reflect changes in diagnostic criteria and the way that these are interpreted, changes in parental perception of impairment, and increased clinician and community awareness of ADHD.^8^ These increases in ADHD medication prescriptions have been particularly pronounced in adults and females. In the US, while the overall prevalence in adults receiving ADHD medication increased from 0.5% to 2.0% from 2001 to 2015,^6^ the prevalence in women aged 15 to 44 years increased from 0.9% in 2003 to 4.0% in 2015.^9^ These significant shifts highlight a critical question: do those receiving ADHD medication today benefit from the treatment in the same way as those who received it a decade ago? Understanding these evolving dynamics is essential for optimizing treatment strategies across diverse patient populations, as well as for informing treatment guidelines and recommendations for both children and adults.

The significant shift in ADHD prescription rates in Sweden presents a unique opportunity for a natural experiment to explore to what extent the effects of ADHD medication vary as prescription prevalence increases within a society. We hypothesize that the effect size of the associations of ADHD medication with self-harm, injuries, traffic crashes, and crime will have decreased, as these medications are now being prescribed to a wider group of patients^10^ that include those with fewer impairments and risky behaviors who may not benefit as much from pharmacotherapy.

## Methods

This study was approved by the Swedish Ethical Review Authority (2020-06540). According to Swedish law, informed consent is not required for pseudo-anonymized register-based research. Our study was reported according to the Reporting of Studies Conducted Using Observational Routinely Collected Health Data-Pharmacoepidemiological Research (RECORD-PE) guidelines.^11^

### Data Sources

We used the following Swedish national registers to identify the study cohort (details in the eMethods in Supplement 1): the Total Population Register, the National Patient Register, the Prescribed Drug Register, the Cause of Death Register, the National Crime Register, and the Longitudinal Integration Database for Health Insurance and Labor Studies.

### Study Cohort and Self-Controlled Case Series Design

**Figure 1.**
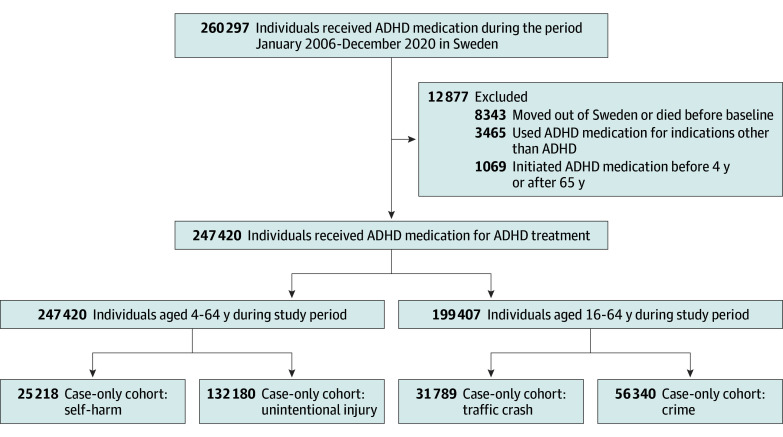
Flowchart of Sample Selection for the Study Cohorts ADHD indicates attention-deficit/hyperactivity disorder.

We identified all individuals aged 4 to 64 years who were prescribed ADHD medication and were alive and residing in Sweden between January 1, 2006, and December 31, 2020. From this base cohort, we further identified 4 case-specific cohorts for self-harm, unintentional injury, traffic crashes, and crime, consisting of individuals who experienced at least 1 relevant event during the study period (Figure 1). Participants were followed up from baseline, defined as January 1, 2006, or their 4th birthday (or 16th birthday for traffic crashes and crime, as these outcomes could only be assessed in individuals older than 16 years), whichever was later, until their 65th birthday, death, emigration, or December 31, 2020, whichever occurred first.

We used a self-controlled case series (SCCS) design^12^ to investigate the association between ADHD medication use and the rate of abovementioned outcomes. In this design, individuals act as their own controls, effectively controlling for both measured and unmeasured confounders, such as genetics, socioeconomic status, or other individual characteristics that remain constant during the follow-up. This is particularly useful in pharmacoepidemiological studies using secondary health care data, where detailed information on potential confounders may be limited. This makes SCCS a powerful method for examining the temporal relationship between exposure and outcomes.^12^

### Measurements

#### Exposure

We identified the following ADHD medications approved in Sweden during the study period: stimulants, methylphenidate (MPH) (Anatomical Therapeutic Chemical [ATC] code N06BA04), amphetamine (ATC code N06BA01), dexamphetamine (ATC code N06BA02), and lisdexamfetamine (ATC code N06BA12) and nonstimulants, atomoxetine (ATC code N06BA09) and guanfacine (ATC code C02AC02). Exposure periods were defined as days covered by dispensed ADHD medication and the length of a treatment period was determined using a validated algorithm.^13^ A gap of 30 days or more between 2 consecutive treatment periods (that is, the interval between the end date of the last treatment period and the start date of the next one) was defined as a nontreatment period. We included dispensation data in 2005 and 2021 to ascertain the treatment status over the first and the last period during the follow-up from 2006 to 2020.

#### Outcomes

The primary outcomes included self-harm, unintentional injury, traffic crashes, and crime, all analyzed as recurrent events. Information on self-harm (*International Statistical Classification of Diseases and Related Health Problems, Tenth Revision [ICD-10]* codes X60-X84 and Y10-Y34), unintentional injury (*ICD-10* codes V01-X59), and traffic crashes (*ICD-10* codes V01-V99) were obtained from the national patient register. To avoid counting the same event multiple times, we included only unplanned visits. For traffic crashes, we also included driving under the influence, identified from the National Crime Register. Crime was defined as any conviction for a crime from the National Crime Register; if the date of the crime was unavailable, the date of conviction was used.^14^

### Statistical Analysis

To explore the association between ADHD medication use and the outcomes, we divided the follow-up time into consecutive periods for each individual. A new period began after a treatment switch (from treatment to nontreatment or vice versa) or a change of any time-varying covariate. Incidence rate ratios (IRRs) were estimated to compare the rate of outcome events during medicated period with the rate during nonmedicated periods in the same individual using conditional Poisson regression, with robust standard errors accounting for the correlated data within individual. The SCCS design implicitly controls for measured and unmeasured time-invariant confounders that differ between individuals. Furthermore, we explicitly adjusted for time-varying covariates, including age per calendar year, concurrent use of other psychotropic medication, including antipsychotics (ATC code N05A); anxiolytics, hypnotics, and sedatives (ATC code N05B or N05C); antidepressants (ATC code N06A); antiepileptic drugs (ATC code N03A), and drugs used in addictive disorders (substance use disorders; ATC code N07B), as well as seasonality in Sweden (school/work season: September to June; or summer holiday season: July to August).

To explore how the associations of ADHD medication with real-world outcomes vary with the prescription prevalence, we examined the associations across 3 time periods: 2006 to 2010, 2011 to 2015, and 2016 to 2020, during which the prevalence of ADHD medication use increased continuously (eFigure 1 in Supplement 1). Whether the estimates were similar or exhibited a trend in associations across the 3 time periods was tested using meta-regression with random-effects models to determine statistical significance over time.^15^ We conducted sex-stratified analyses for all outcomes. Age-stratified analyses were performed for self-harm and injury subcohorts (younger than 18 years and 18 years or older), but not for traffic crashes or criminality, as these outcomes could only be defined in adults.

#### Sensitivity Analyses

Given the increasing proportion of adults and females prescribed ADHD medication between 2006 and 2020, we conducted an age-matched and sex-matched analysis, with ADHD medication users in the latter 2 periods matched to those in the first period (2006 to 2010). This approach examined whether any changes in the associations of ADHD medication with real-world outcomes over time were influenced by shifts in the age and sex distribution of those treated. We used a matching method with replacement and applied cluster robust standard errors in our statistical analyses to account for repeated observations of the same individuals over time. We also repeated the main analysis with only MPH users to investigate how changes in ADHD medication types over time might affect our patterns of results, as other ADHD medications, for example, lisdexamfetamine, have been increasingly used in recent years.

Data management was performed using SAS version 9.4 (SAS Institute). Analyses were conducted with R version 4.3.1 (R Foundation for Statistical Computing).

## Results

In total, there were 247 420 ADHD medication users aged 4 to 64 years (99 361 females [40.2%] and 148 059 males [59.8%]) identified during the study period. These individuals were distributed across the 3 prespecified time frames as follows (Table 1): 57 263 for the period 2006 to 2010 (33.9% female; median age, 16 years), 127 241 for 2011 to 2015 (38.2% female; median age, 17 years), and 200 141 for 2016 to 2020 (41.6% female; median age, 18 years). Both the median age and the proportion of female ADHD medication users have increased over time, and proportion of ADHD medication users experiencing self-harm, traffic crashes, and crime showed a slight decline (eFigure 2 in Supplement 1). For the SCCS analyses (Figure 1), 25 218 individuals with at least 1 event of self-harm, 132 180 with at least 1 event of unintentional injury, 31 789 with at least 1 traffic crash, and 56 340 with at least 1 event of crime during the study period were included.

**Table 1.** Characteristics of Attention-Deficit/Hyperactivity Disorder (ADHD) Medication Users in Sweden From 2006 to 2020

Characteristic	2006-2010	2011-2015	2016-2020
Total No.	57 263	127 241	200 141
Age at baseline, y, median (IQR)	16 (11-28)	17 (12-30)	18 (12-31)
Sex, No. (%)			
Female	19 403 (33.88)	48 563 (38.17)	83 197 (41.57)
Male	37 860 (66.12)	78 678 (61.83)	116 944 (58.43)
Number of ADHD medication users with at least 1 event, No. (%)			
Self-harm	4329 (7.56)	7725 (6.07)	9520 (4.76)
Unintentional injury	17 089 (29.84)	40 551 (31.87)	56 989 (28.47)
Traffic crashes^a^	5871 (10.25)	11 173 (8.78)	15 622 (7.81)
Crime^a^	12 436 (21.72)	21 797 (17.13)	25 764 (12.87)

^a^ Number of ADHD medication users with at least 1 event (%) is calculated among those aged 16 years and older.

**Figure 2.**
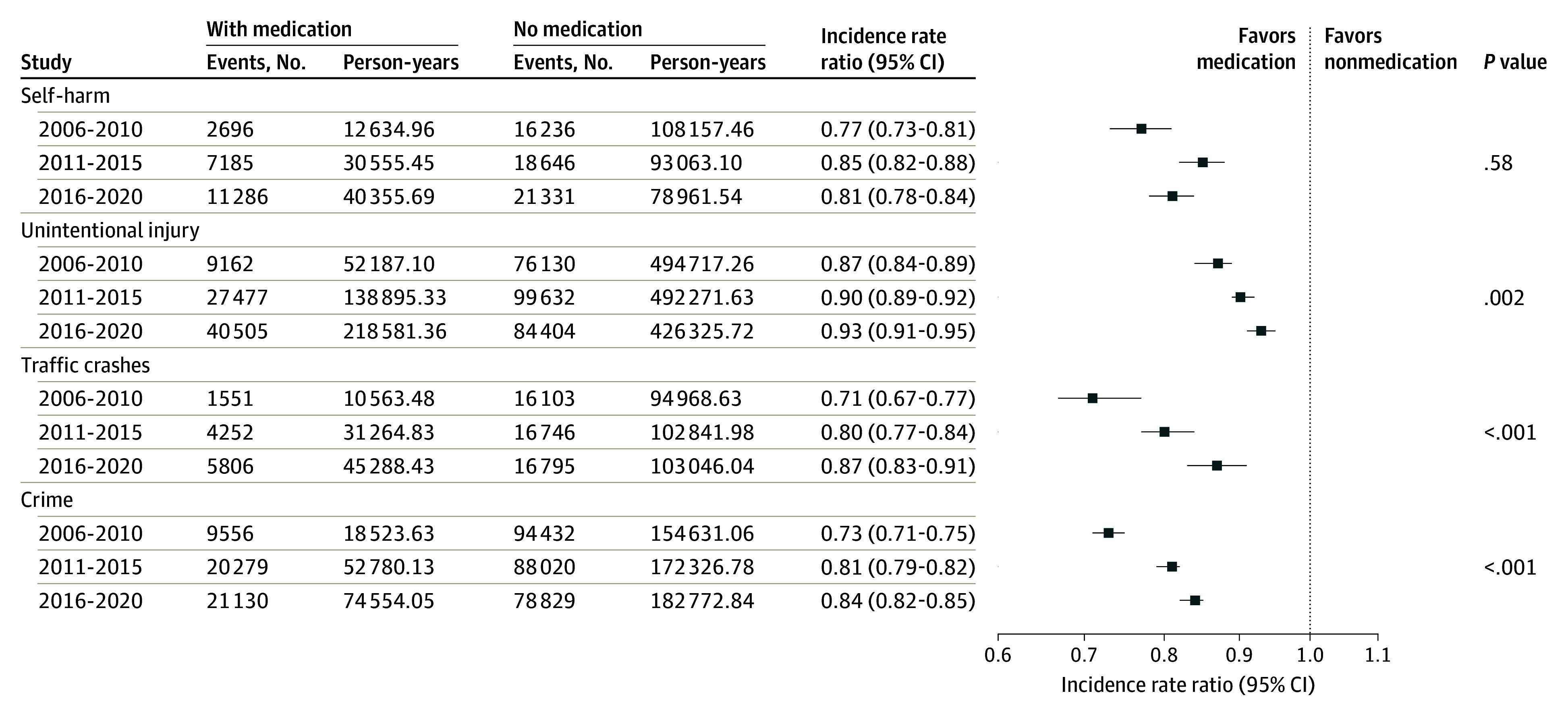
Within-Individual Association Between Attention-Deficit/Hyperactivity Disorder Medication Use and Real-World Outcomes

The prescription prevalence increased from on average 0.4% during 2006 to 2010, 1.0% during 2011 to 2015, to 1.6% during 2016 to 2020 (from 0.8% to 2.4% in children and from 0.2% to 1.1% in adults) (eFigure 1 in Supplement 1). ADHD medication use was associated with statistically significant lower rates of all studied outcomes throughout the study period, even when the rate of prescriptions increased nearly 5-fold in the population (Figure 2). For self-harm, the association was strongest during 2006 to 2010 (IRR, 0.77; 95% CI, 0.73-0.81) and slightly attenuated in the 2 recent periods. The change was not statistically significant (*P* value for trend = .58). In contrast, there was a statistically significant decreasing trend of IRRs for unintentional injury (*P* value for trend < .01), with the strongest association in 2006-2010 (IRR, 0.87; 95% CI, 0.84-0.89) and decreasing over time (for 2011 to 2015; IRR, 0.90; 95% CI, 0.89-0.92; for 2016 to 2020; IRR, 0.93; 95% CI, 0.91-0.95). Similar patterns were found for traffic crashes (IRR changed from 0.71, 95% CI, 0.67-0.77; to IRR, 0.87; 95% CI, 0.83-0.91) and crime (IRR changed from 0.73; 95% CI, 0.71-0.75; to IRR, 0.84; 95% CI, 0.82-0.85), with statistically significant decreasing trends over time (*P* value for trend < .01).

**Figure 3.**
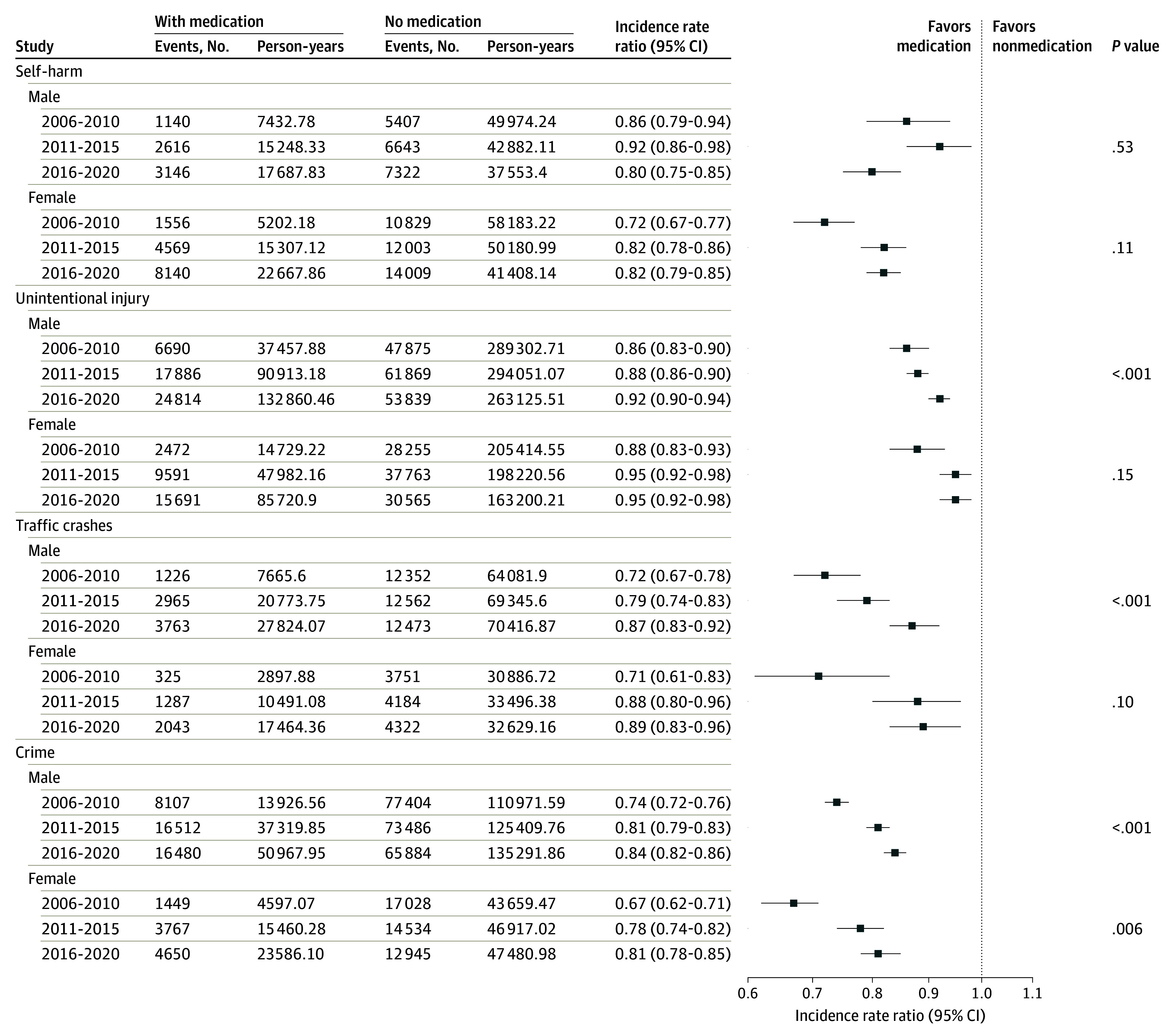
Within-Individual Association Between Attention-Deficit/Hyperactivity Disorder Medication Use and Real-World Outcomes by Sex

In sex-stratified analyses, it was found that the use of ADHD medication was statistically significantly associated with lower rates of all studied outcomes in both males and females throughout the study period. Both sexes exhibited a trend of decreasing effect size over time for the studied outcomes, with the strongest association (except for unintentional injury) consistently observed in females during 2006 to 2010, compared with later years (Figure 3). Additionally, age-stratified analysis indicated that ADHD medication use was associated with a reduced risk of self-harm and unintentional injury in both children and adults (eFigure 3 in Supplement 1). For unintentional injuries, significant attenuation of the association was only observed in adults but not in children.

The results of the sensitivity analyses are summarized in Table 2. Sample characteristics of the age-matched and sex-matched cohort are detailed in eTable 1 in Supplement 1, and the results largely aligned with the main analyses, although the trend of the association over time was not statistically significant for crime. When the analysis was limited to MPH users, the results remained similar to the main analyses. These results suggest that changes in the demographic distribution of individuals receiving ADHD medication and the type of medication do not fully account for the weakening trend of observed associations over time.

**Table 2.** Summary Results of Sensitivity Analyses

	With medication		Without medication		IRR (95% CI)	*P* value for trend
	No. of events	Person-years	No. of events	Person-years		
**Age-matched and sex-matched cohorts**						
Self-harm						
2006-2010	6968	29 514.62	2695	12 609.92	0.79 (0.75-0.84)	.58
2011-2015	5495	25 060.61	3558	16 905.07	0.95 (0.90-1.00)	
2016-2020	7877	23 664.08	5671	18 235.17	0.80 (0.77-0.84)	
Unintentional injury						
2006-2010	20 406	110 933.19	9162	52 187.10	0.88 (0.86-0.92)	<.01
2011-2015	20 553	97 825.91	12 686	65 357.60	0.92 (0.89-0.94)	
2016-2020	20 120	93 042.84	13 245	70 317.80	0.94 (0.91-0.97)	
Traffic crashes						
2006-2010	5910	25 780.87	1551	10 563.48	0.74 (0.69-0.80)	<.01
2011-2015	4206	23 498.67	1973	14 579.89	0.83 (0.77-0.89)	
2016-2020	4949	23 561.26	2808	16 010.54	0.88 (0.83-0.94)	
Crime						
2006-2010	38 593	44 445.62	9556	18 523.63	0.75 (0.73-0.77)	.65
2011-2015	25 251	41 597.08	9503	24 239.52	0.80 (0.78-0.82)	
2016-2020	21 772	41 266.95	9054	26 884.86	0.77 (0.75-0.80)	
**Only methylphenidate users**						
Self-harm						
2006-2010	14 666	97 297.95	2330	10 985.77	0.75 (0.71-0.80)	.13
2011-2015	17 180	85 133.29	5767	25 816.64	0.81 (0.78-0.85)	
2016-2020	23 467	85 866.10	5188	21 286.78	0.81 (0.78-0.84)	
Unintentional injury						
2006-2010	70 107	453 683.26	7925	45 582.17	0.86 (0.83-0.89)	.03
2011-2015	93 415	460 535.36	23 265	118 960.58	0.90 (0.89-0.92)	
2016-2020	90 743	464 350.19	23 288	128 616.90	0.92 (0.90-0.93)	
Traffic crashes						
2006-2010	14 169	82 781.77	1331	9229.02	0.70 (0.65-0.75)	.01
2011-2015	14 760	91 478.36	3681	26 796.08	0.83 (0.79-0.87)	
2016-2020	16 908	107 287.34	3047	24 613.83	0.86 (0.81-0.91)	
Crime						
2006-2010	82 752	135 492.52	8332	16 070.73	0.72 (0.70-0.74)	.08
2011-2015	76 907	154 369.60	17 386	44 972.96	0.81 (0.79-0.83)	
2016-2020	77 855	190 958.57	9920	39 016.83	0.81 (0.79-0.83)	

Abbreviation: IRR, incidence rate ratio.

## Discussion

In this longitudinal population-based study of 247 420 individuals using ADHD medication between 2006 and 2020, we consistently found ADHD medication to be associated with lower rates of self-harm, unintentional injury, traffic crashes, and crime across all analyzed time periods, age groups, and sexes. However, magnitude of associations between ADHD medication use and lower risk of unintentional injury, traffic crashes, and crime appear to have attenuated over time, coinciding with an increase in prescription prevalence during the same period. The weakening trends for unintentional injury and traffic crashes were not fully explained by changes in age and sex distribution of the medication users, whereas the trend for crime was no longer statistically significant. These findings suggest that the declining strength of the associations of ADHD medication and real-world outcomes could be attributed to the expansion of prescriptions to a broader group of individuals having fewer symptoms or impairments.^10^

To our knowledge, no prior studies have examined the changes in the effectiveness of ADHD medications or their associations with real-world outcomes over time. Our findings are consistent with previous population-based studies that have demonstrated protective associations of ADHD medication on these downstream consequences of ADHD symptoms.^2,16^ We now show that these associations remain even when prescription prevalence increased substantially. With increasing number of ADHD diagnoses and medication prescriptions, it is likely that the people being diagnosed and treated for ADHD today will have presented with less severe ADHD, showing less symptoms or fewer impairments than those who were diagnosed and treated 15 years ago.^10^ Even though the magnitude of associations between ADHD medication use and risk of these more distal outcomes appears to decrease as the rates of prescription increase, there are still clear benefits associated with ADHD medication use. However, ADHD medications, particularly stimulants, are also associated with adverse effects, such as reduced appetite, delayed growth, insomnia, and increased heart rate or blood pressure.^17^ These results remind us that in clinical practice, the treatment decisions should be made by a careful balancing of the benefits and risks for each individual. Future research is needed to identify whether there are subgroups of patients who may benefit more from alternative or supplemental treatment strategies. If rates of prescribing in countries like Sweden continue to increase, and in countries like the US where for some age-groups and regions rates of prescribing already exceed epidemiological prevalence,^4,7^ the need to balance benefit and risk is even more important. In these situations, the proportion of people who are being treated with ADHD medication but who have a subsyndromal presentation will increase.^18^ Unfortunately, there is limited evidence on the risks and benefits of ADHD medications in subsyndromal ADHD and so further research is required.

Interestingly, the strongest associations between ADHD medication and real-world outcomes were consistently observed in females during the 2006 to 2010 period. We believe that this probably reflects differences in recognition of ADHD between males and females at this time, resulting in the diagnosis and treatment of only those females with the most severe symptoms.^19^ As the sex differences in recognition of ADHD have reduced over time, reflected by an increasing proportion of women in the samples, the sex differences on the various real-world outcomes have also narrowed. Notably, self-harm was the only outcome with a higher absolute number of events in females than in males. This is not surprising, as it is well recognized that females with ADHD are more likely to have an inattentive presentation of ADHD and also to exhibit internalizing rather than externalizing behaviors.^19^ Outcomes, such as unintentional injuries, traffic crashes, and crime, are more likely to be associated with impulsivity and externalizing behaviors.^19^

While the rising trends in ADHD diagnoses and medication prescriptions are well recognized, this is the first study to examine how these changes influence the associations of ADHD medication and serious real-world outcomes over time. As ADHD diagnosis and medication prescription rates rise, the treatment guidelines should be regularly reviewed to reflect changing patient population. However, more high-quality research is needed to inform these updates, and our findings provide an important foundation for future work. Specifically, studies should explore the potential factors contributing to the declining effect sizes, including changes to ADHD diagnostic criteria and prescribing guidelines, variations in medication adherence, and shifts in public health interventions targeting injury prevention and mental health. Additionally, research should explore individual variations in medication effects across ADHD severity, comorbidities, and treatment adherence to enhance clinical recommendations. Incorporating patient-reported outcomes through longitudinal surveys may also provide valuable insights into individuals’ perceived benefits of ADHD medication over time. Lastly, examining complementary interventions—such as behavioral therapy alone or in combination with pharmacotherapy—may further optimize treatment outcomes.

### Strength and Limitations

The strengths of this study include a national sample comprising both children and adults, a long follow-up period, validated measures of exposure and outcome, and the use of SCCS design. However, there are several limitations to consider. First, due to the observational nature, our results cannot establish causal effects of ADHD medication treatment on the outcomes. Unmeasured time-varying confounders, such as lifestyle factors associated with initiating and stopping pharmacotherapy, which may also influence their risk of a certain outcome, could contribute to the observed associations. Second, we cannot rule out exposure misclassification, as some individuals may not have consistently adhered to their prescribed medication, leading to potential underestimation of the true effects. Third, the findings are based on Swedish population data, and generalizations across cultures and countries should be made with caution. Fourth, these outcomes represent only a subset of those associated with ADHD that we would expect ADHD medications to affect. We cannot draw conclusions about ADHD symptoms or other outcomes.

## Conclusions

While ADHD medications are consistently associated with reduced risk of serious real-world outcomes, the magnitude of these associations have decreased over time alongside rising prescription rates. This underscores the importance of continuously evaluating medication use in different patient populations. Further research is needed to better understand individual-level treatment effects based on patient characteristics, ensuring that clinical decision-making remains well informed.

## Notes

Supplement 1. eMethods **eTable 1.** Characteristics of ADHD medication users from 2006 to 2020 in age- and sex-matched cohort. **eFigure 1.** The prevalence of ADHD medication users in Sweden from 2006 to 2020. **eFigure 2.** Proportion of ADHD medication users with the outcomes of interest over time. **eFigure 3.** Within-individual association between ADHD medication use and real-world outcomes among children and adults.

Supplement 2. Data sharing statement
