# Supplementary material for: Increased Prescribing of Attention-Deficit/Hyperactivity Disorder Medication and Real-World Outcomes Over Time
Source: JAMA Psychiatry. 2025 Jun 25;82(8):830–7. doi: 10.1001/jamapsychiatry.2025.1281 (PMC12199179; doi:10.1001/jamapsychiatry.2025.1281)
Supplement: Supplement 1. — eMethods eTable 1. Characteristics of ADHD medication users from 2006 to 2020 in age- and sex-matched cohort. eFigure 1. The prevalence of ADHD medication users in Sweden from 2006 to 2020. eFigure 2. Proportion of ADHD medication users with the outcomes of interest over time. eFigure 3. Within-individual association between ADHD medication use and real-world outcomes among children and adults. [file jamapsychiatry-e251281-s001.pdf]

Supplemental Online Content

Li L, Coghill D, Sjölander A, et al. Increased prescribing of ADHD medication and real-world outcomes over time. *JAMA Psychiatry*. Published online June 25, 2025.
doi:10.1001/jamapsychiatry.2025.1281

eMethods

eTable 1. Characteristics of ADHD medication users from 2006 to 2020 in age- and sex-matched cohort.

eFigure 1. The prevalence of ADHD medication users in Sweden from 2006 to 2020.

eFigure 2. Proportion of ADHD medication users with the outcomes of interest over time.

eFigure 3. Within-individual association between ADHD medication use and real-world outcomes among children and adults.

This supplemental material has been provided by the authors to give readers additional information about their work.

© 2025 Li L et al. *JAMA Psychiatry*.

eMethods

Data sources

Data were obtained by linking multiple Swedish registries using the unique personal identification number assigned to all residents in Sweden. The Total Population Register(1) includes all individuals in Sweden born since 1932, who were alive in 1963 and later. It also contains information on all migrations in or out of Sweden since 1969. The National Patient Register(2) contains data on inpatient care since 1973 and outpatient care since 2001, with diagnosis of diseases recorded by the Swedish version of the International Statistical Classification of Diseases and Related Health Problems, Tenth Revision (ICD-10) since 1997. The Prescribed Drug Register(3) includes detailed information on all dispensed drugs in Sweden, coded according to Anatomical Therapeutic Chemical (ATC) classification, since 1 July 2005. The Cause of Death Register(4) contains information on all registered deaths since 1961, including underlying and contributing causes of death. The National Crime Register includes detailed information about all criminal convictions since 1973;(5) The Longitudinal Integration Database for Health Insurance and Labor Studies(6) covers the entire Swedish population aged 16 or older since 1990.

References:

1. Ludvigsson JF, Almqvist C, Bonamy A-KE, Ljung R, Michaëlsson K, Neovius M, et al. Registers of the Swedish total population and their use in medical research. European Journal of Epidemiology. 2016;31(2):125-36.
2. Ludvigsson JF, Andersson E, Ekbom A, Feychting M, Kim J-L, Reuterwall C, et al. External review and validation of the Swedish national inpatient register. BMC Public Health. 2011;11(1):450.
3. Wettermark B, Hammar N, Fored CM, Leimanis A, Otterblad Olausson P, Bergman U, et al. The new Swedish Prescribed Drug Register--opportunities for pharmacoepidemiological research and experience from the first six months. Pharmacoepidemiol Drug Saf. 2007;16(7):726-35.
4. Brooke HL, Talbäck M, Hörnblad J, Johansson LA, Ludvigsson JF, Druid H, et al. The Swedish cause of death register. European Journal of Epidemiology. 2017;32(9):765-73.
5. Lichtenstein P, Halldner L, Zetterqvist J, Sjölander A, Serlachius E, Fazel S, et al. Medication for attention deficit–hyperactivity disorder and criminality. New England Journal of Medicine. 2012;367(21):2006-14.

© 2025 Li L et al. *JAMA Psychiatry*.

6. Ludvigsson JF, Svedberg P, Olén O, Bruze G, Neovius M. The longitudinal integrated database for health insurance and labour market studies (LISA) and its use in medical research. Eur J Epidemiol. 2019;34(4):423-37.

eTable 1 Characteristics of ADHD medication users from 2006 to 2020 in age- and sex-matched cohort.

	Total	2006-2010	2011-2015	2016-2020
**Self-harm cohort**				
N	25,473	8,491	8,491	8,491
Median age at baseline (IQR)	20 (16,32)	20 (16,32)	20 (16,32)	20 (16,32)
Male (%)	14,160 (55.59)	4,720 (55.59)	4,720 (55.59)	4,720 (55.59)
**Injury cohort**				
N	98,946	32,982	32,982	32,982
Median age at baseline (IQR)	16 (12,27)	16 (12,27)	16 (12,27)	16 (12,27)
Male (%)	67,779(68.50)	22,593(68.50)	22,593(68.50)	22,593(68.50)
**Traffic cohort**				
N	26,034	8,678	8,678	8,678
Median age at baseline (IQR)	24 (16,36)	24 (16,36)	24 (16,36)	24 (16,36)
Male (%)	18,909 (72.63)	6,303 (72.63)	6,303 (72.63)	6,303 (72.63)
**Crime cohort**				
N	45,840	15,280	15,280	15,280
Median age at baseline (IQR)	22 (16,34)	22 (16,34)	22 (16,34)	22 (16,34)
Male (%)	34,449(75.15)	11,483(75.15)	11,483(75.15)	11,483(75.15)

IQR: The interquartile range

© 2025 Li L et al. *JAMA Psychiatry*.

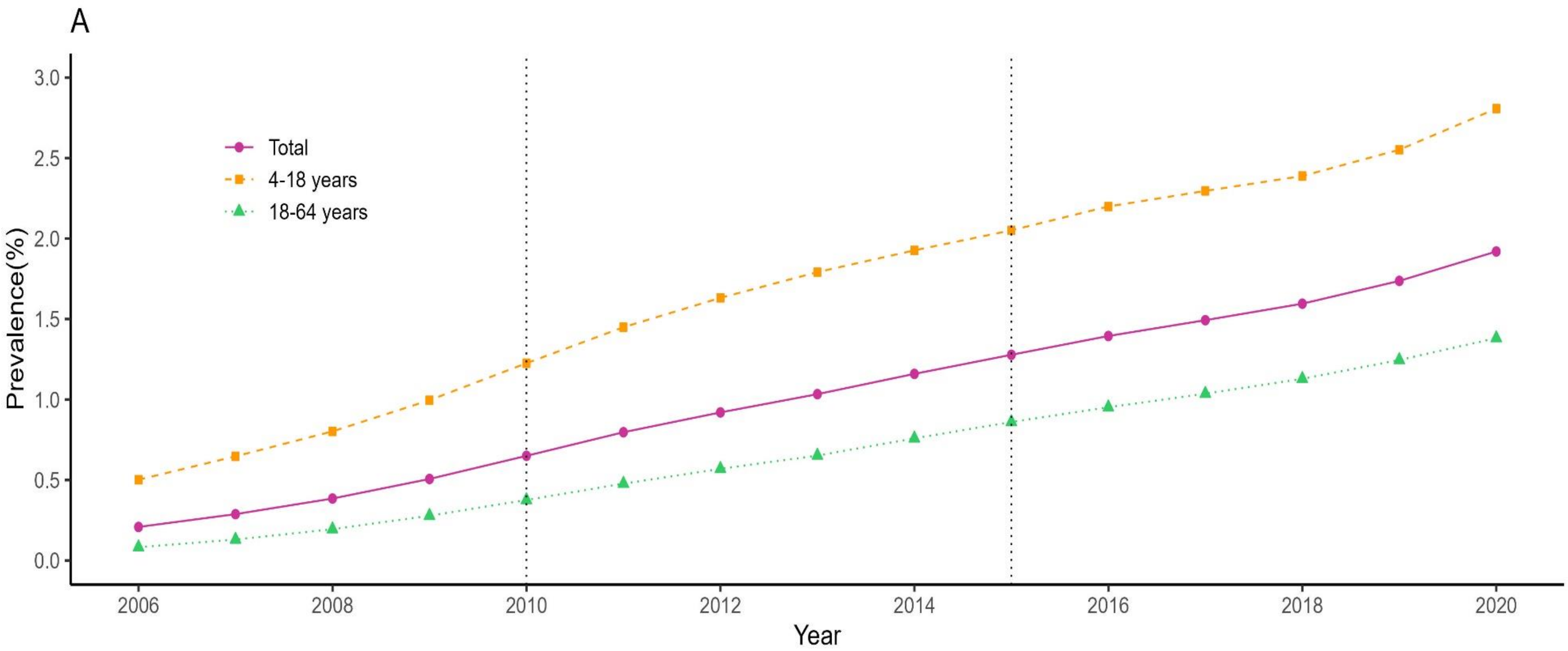

eFigure 1. The prevalence of ADHD medication users in Sweden from 2006 to 2020.

© 2025 Li L et al. *JAMA Psychiatry*.

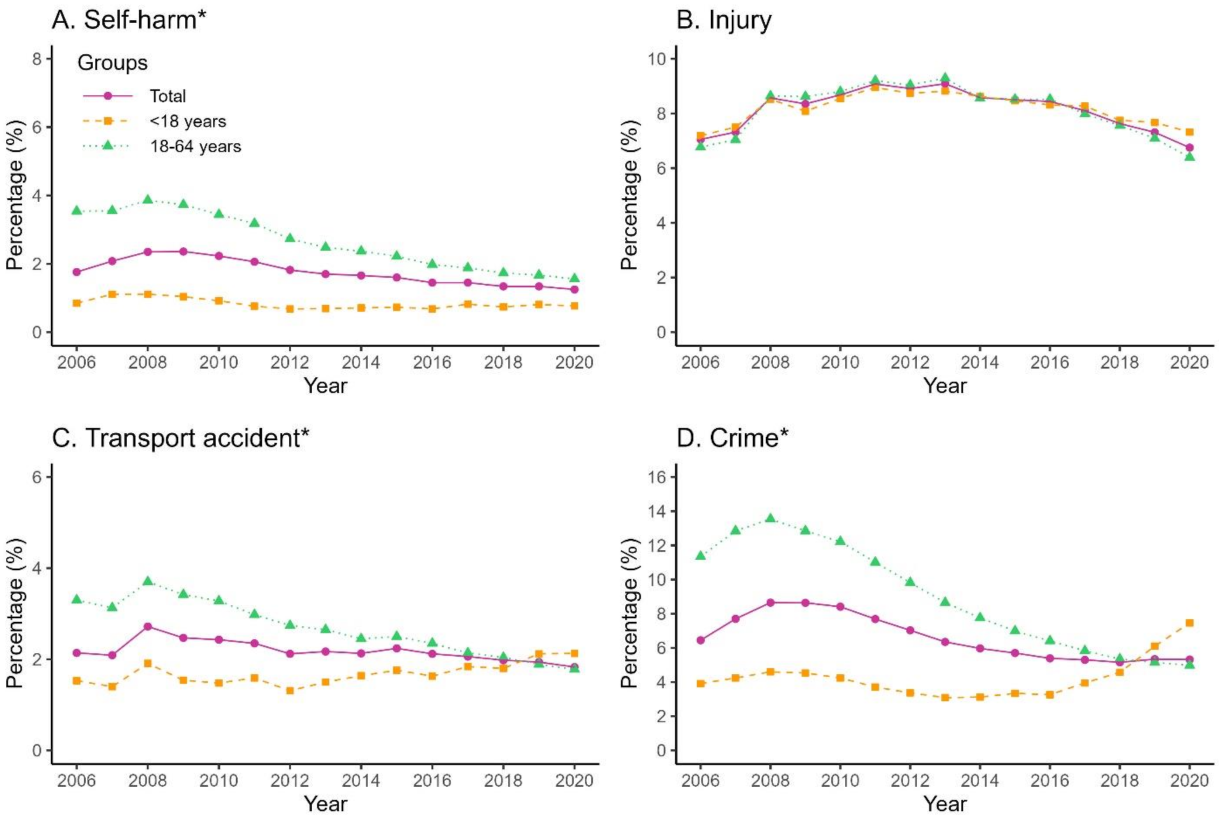

eFigure 2. Proportion of ADHD medication users with the outcomes of interest over time.

The proportion (%) is calculated as the number of ADHD medication users with outcomes divided by the total number of ADHD medication users per year. For Parts C and D, the <18 years group includes only individuals aged 15-17.

* Statistically significant temporal trend ($p < 0.05$).

© 2025 Li L et al. *JAMA Psychiatry*.

	With medication No. of events	With medication Person-years	No medication No. of events	No medic Person-y	Incidence rate ratio		
**Self-harm**							
**4-18 years**							
2006-2010	677	5797.26	3812	52735.82	0.82 (0.73-0.93)		
2011-2015	1279	8407.71	3350	28742.67	0.84 (0.77-0.93)		
2016-2020	2658	6517.03	3594	10601.07	0.79 (0.74-0.85)		P=0.44
**18-64 years**							
2006-2010	2019	6837.7	12424	55421.64	0.78 (0.73-0.83)		
2011-2015	5906	22147.73	15296	64320.43	0.85 (0.82-0.89)		
2016-2020	8628	33838.66	17737	68360.48	0.84 (0.81-0.87)		P=0.23
**Accidental injury**							
**4-18 years**							
2006-2010	5348	32142.84	41742	289756.02	0.84 (0.81-0.88)		
2011-2015	12982	68255.36	48391	242435.52	0.87 (0.84-0.90)		
2016-2020	17556	88914.89	29522	131245.87	0.88 (0.85-0.90)		P=0.08
**18-64 years**							
2006-2010	3814	20044.26	34388	204961.24	0.89 (0.85-0.93)		
2011-2015	14495	70639.97	51241	249836.11	0.94 (0.91-0.96)		
2016-2020	22949	129666.47	54882	295079.85	0.98 (0.96-1.00)		P< 0.01

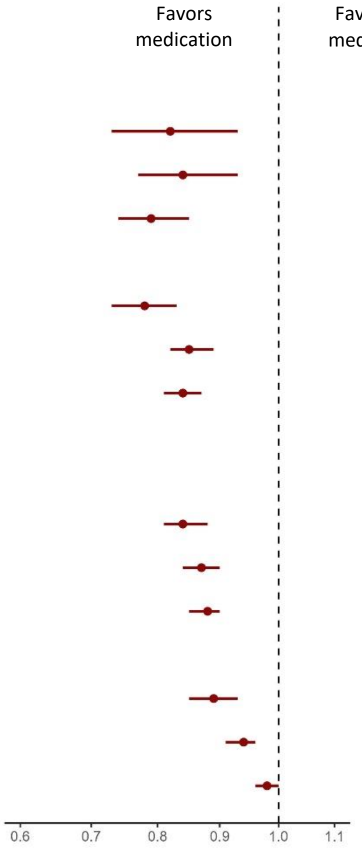

eFigure 3. Within-individual association between ADHD medication use and real-world outcomes among children and adults.

© 2025 Li L et al. *JAMA Psychiatry*.
