# Supplementary material for: Increased Prescribing of Attention-Deficit/Hyperactivity Disorder Medication and Real-World Outcomes Over Time
Source: JAMA Psychiatry. 2025 Jun 25;82(8):830–7. doi: 10.1001/jamapsychiatry.2025.1281 (PMC12199179; doi:10.1001/jamapsychiatry.2025.1281)
Supplement: Supplement 2. — Data sharing statement [file jamapsychiatry-e251281-s002.pdf]

Data Sharing Statement

Li. Increased Prescribing of Attention-Deficit/Hyperactivity Disorder Medication and Real-World Outcomes Over Time. *JAMA Psychiatry*. Published June 25, 2025.
doi:10.1001/jamapsychiatry.2025.1281

Data
Data available: No

Additional Information
Explanation for why data not available: The data supporting the findings of this study are available from Statistics Sweden and The Swedish National Board of Health and Welfare; however, due to ethical permissions and restrictions, these data are not publicly available.
